# Determining the level of insomnia in postpartum women, comparing their age and child’s age

**DOI:** 10.1192/j.eurpsy.2025.2392

**Published:** 2025-08-26

**Authors:** P. Batchuluun, P. Sarantuya, T. Urtnasan, S. Surlegbaatar

**Affiliations:** 1 Medical Department; 2 Basic medical department, Etugen University, Ulaanbaatar, Mongolia

## Abstract

**Introduction:**

Studies have shown that postpartum women are more affected by sleep disorders than women who have not given birth. Reasons for sleep disturbances include insufficient sleep time, poor sleep quality, and postpartum depression. Having a sleep disorder has a negative effect on the formation of a close relationship between mother and child.

**Objectives:**

To determine the level of insomnia in postpartum women and study the correlation.

**Methods:**

The study will be conducted by women who agreed to sleep disorder detection questionnaires specially prepared by the World Health Organization for doctors in primary health care institutions. The results of the research parameters were statistically processed using Microsoft Word 2016, Microsoft Excel 2016, and SPSS 26 programs.

**Results:**

Of the 100 women who participated in the study, 25% had no insomnia, 48% had mild sleep disorders, 27% had sleep disturbances, 23% had no stress, 42% had moderate stress, and 35% had high stress. 16% of women aged 20-24 have no insomnia, 72% have mild sleep disorders, and 12% have sleep disorders. 23.8% of women aged 25-29 have no insomnia, 47.6% have mild sleep disorders, and 28.6% have sleep disorders. 25% of women aged 30-34 have no insomnia, 40% have mild sleep disorders, and 35% have sleep disorders. 45% of women aged 35-39 have no insomnia, 20% have mild sleep disorders, and 35% have sleep disorders. 0% of women aged 40-44 have no insomnia, 70% have mild sleep disorders, and 30% have sleep disorders. 50% of women aged 45-49 have no insomnia, 25% have mild sleep disorders, and 25% have sleep disorders. 35% of women with children aged 0-3 months have no insomnia, 35% have mild sleep disorders, and 30% have sleep disorders. 15% of women with children aged 4-6 months have no insomnia, 50% have mild sleep disorders, and 35% have sleep disorders. 35% of women with children aged 7-9 months have no insomnia, 45% have mild sleep disorders, and 20% have sleep disorders. 15% of women with children aged 10 months to 1 year have no insomnia, 50% have mild sleep disorders, and 35% have sleep disorders. 25% of women with 2-year-old children have no insomnia, 60% have mild sleep disorders, and 15% have sleep disturbances.

**Conclusions:**

As mothers age, insomnia rates increase, stress levels decrease, and postpartum depression rates increase. As children age, sleep deprivation rates decrease, stress levels decrease, and postpartum depression rates decrease. As maternal fertility increases, insomnia rates decrease, stress levels decrease, and postpartum depression rates decrease. Insomnia, stress, and postpartum depression are also affected by living conditions.

**Disclosure of Interest:**

None Declared

